# Epidemiological shifts in 23 acute infectious diseases in Southwest China (2005–2024): trends and implications for surveillance

**DOI:** 10.1186/s12889-025-24390-9

**Published:** 2025-09-24

**Authors:** Runyou Liu, Yichun Liu, Qiang Lv, Yajia Lan

**Affiliations:** 1 Sichuan Center for Disease Control and Prevention, Chengdu, 610041 Sichuan P.R. China; 2 West China School of Public Health and West China Fourth Hospital, Sichuan University, Chengdu, 610041 Sichuan P.R. China

**Keywords:** Epidemiological shifts, Acute infectious disease, Surveillance, Trends

## Abstract

**Background:**

Infectious diseases remain a major global health challenge. However, long-term trends of acute infectious diseases in Sichuan Province, an area in southwest China with a high disease burden, are limited. This study examines trends in 23 acute infectious diseases over two decades to provide evidence for evaluating prevention strategies and informing future preparedness efforts.

**Methods:**

We obtained disease incidence and demographic data from the China Information System for Disease Control and Prevention and the National Bureau of Statistics. We analyzed 23 notifiable acute infectious diseases in Sichuan Province from 2005 to 2024, stratifying patterns by sex, age, region, and season. We used Joinpoint regression to assess temporal trends and calculate the average annual percentage change (AAPC). We evaluated spatial trends via trend surface analysis in ArcGIS 10.7.0. Statistical analyses were performed using R 4.4.1 and the Joinpoint Regression Program 5.4.0, setting statistical significance at *p* < 0.05.

**Results:**

From 2005 to 2024, Sichuan recorded incidence and mortality rates of 305.78 and 0.13 per 100,000 people, respectively, for the 23 acute infectious diseases. Excluding hand, foot, and mouth disease and seasonal influenza, the overall incidence rate of the remaining 21 diseases increased (AAPC: 3.4%, 95% CI: 1.75–4.51), with sharp increases occurring from 2022 to 2024. During the study period, the incidence rate increased for 8 diseases, declined for 12 diseases, and remained stable for 3 diseases. Males had a higher incidence rate than females (1.17:1), particularly for zoonotic and vector-borne diseases. The highest incidence rate was among children aged 0–9 years (1,772.04 per 100,000 people). In the first and the last decades, there was a great alteration in dynamic spatial patterns. The north-south trend shifted from a U-shaped pattern in the first decade to a linear pattern in the last decade, while the east-west pattern reversed.

**Conclusions:**

Acute infectious disease patterns in Sichuan have continuously shifted due to evolving prevention strategies and epidemiological trends. Priority diseases requiring enhanced surveillance include seasonal influenza, pertussis, infectious diarrhea, hepatitis E, brucellosis, typhus, and dengue fever. Reducing the disease burden effectively requires targeted strategies that integrate seasonal and regional patterns and intensify surveillance during high-risk periods.

**Supplementary Information:**

The online version contains supplementary material available at 10.1186/s12889-025-24390-9.

## Introduction

Infectious diseases remain among the world’s most pressing health challenges, consistently ranking among the primary causes of mortality and morbidity, particularly among vulnerable populations such as children and the elderly in low- and middle-income countries (LMICs) [1]. Of the more than 70 infectious diseases that are subject to global surveillance, acute infectious diseases — including SARS-CoV-2, mpox(monkeypox), and dengue fever — have the broadest geographic impact. These diseases contribute significantly to mortality while posing serious threats to global economic stability and health security [2]. Recent diseases outbreaks, including the Middle East Respiratory Syndrome (MERS) epidemic, the Ebola outbreak in West Africa, and the Zika virus epidemic, have exposed the fragility of global health systems and presented significant challenges to international disease control efforts [3]. The ongoing Coronavirus disease 2019 (Covid-19) pandemic provides the most striking illustration of this vulnerability. During its first two years, the pandemic caused a global decline in life expectancy of 1.6 years, underscoring its unprecedented impact [4]. These recurring events highlight the persistent threat posed by emerging acute infectious diseases, which continue to challenge global public health due to their unpredictability and rapid spread [5].

Understanding the epidemic patterns and temporal dynamics of infectious diseases is essential for developing effective prevention and control strategies [6]. Several studies have examined these patterns and variations in trends using stable surveillance data from the China Information System for Disease Control and Prevention (CISDCP). For instance, Yang SG et al. observed an initial surge in notifiable infectious disease cases after the SARS outbreak, followed by a decline and subsequent stabilization [7]. Similarly, Du Min et al. documented a decline in Class A and B diseases since 2009, although considerable regional and demographic disparities persist [8]. Additionally, Li ZJ et al. underscored the necessity for prevention strategies that are tailored to geographical, demographic, and seasonal variations by a nationwide prospective surveillance of all-age patients with acute respiratory infections between 2009‒2019 [9]. While these studies provide valuable insights for national policy development, China’s vast territory, diverse climates, and heterogeneous population lifestyles limit the applicability of these insights to precise public health strategies at the provincial or regional levels [10–12].

Sichuan Province, the most populous region in southwest China, with 83.71 million residents across 21 prefecture-level cities and 183 counties, presents a compelling case study for infectious disease surveillance [13]. The province’s complex topography, climatic variations, diverse ethnic composition, and high population mobility contribute to its substantial infectious disease burden [14, 15]. Sichuan has consistently ranked among China’s southwest regions with highest disease incidence, including notable cases such as China’s first reported human H5N6 avian influenza case in 2014 and a significant Lassa fever outbreak in 2024 [16, 17]. However, most existing research on Sichuan has focused primarily on individual pathogens or short-term analyses, resulting in limited understanding of long-term epidemiological trends [18, 19].

To address this gap, our study systematically examines the incidence and mortality rates of 23 major acute infectious diseases in Sichuan from 2005 to 2024 using national surveillance data. This study has three primary objectives: first, to characterize the epidemiological patterns and spatiotemporal trends of these diseases across age groups, genders, and geographic locations; second, to analyze changes in disease transmission patterns and identify diseases requiring priority attention; and third, to develop evidence-based prevention strategies for acute infectious diseases in southwest China.

## Methods

### Data source

Data of incidence rates, mortality rates, number of cases, and deaths stratified by sex, age groups, and geographic location were available from the CISDCP, a web-based, real-time reporting system established in 2004 in response to the SARS epidemic [11]. Annual population data for 2005–2024 were obtained from the Chinese National Bureau of Statistics website.

### Study design

#### Disease classification refinement and selection

**Fig. 1:**
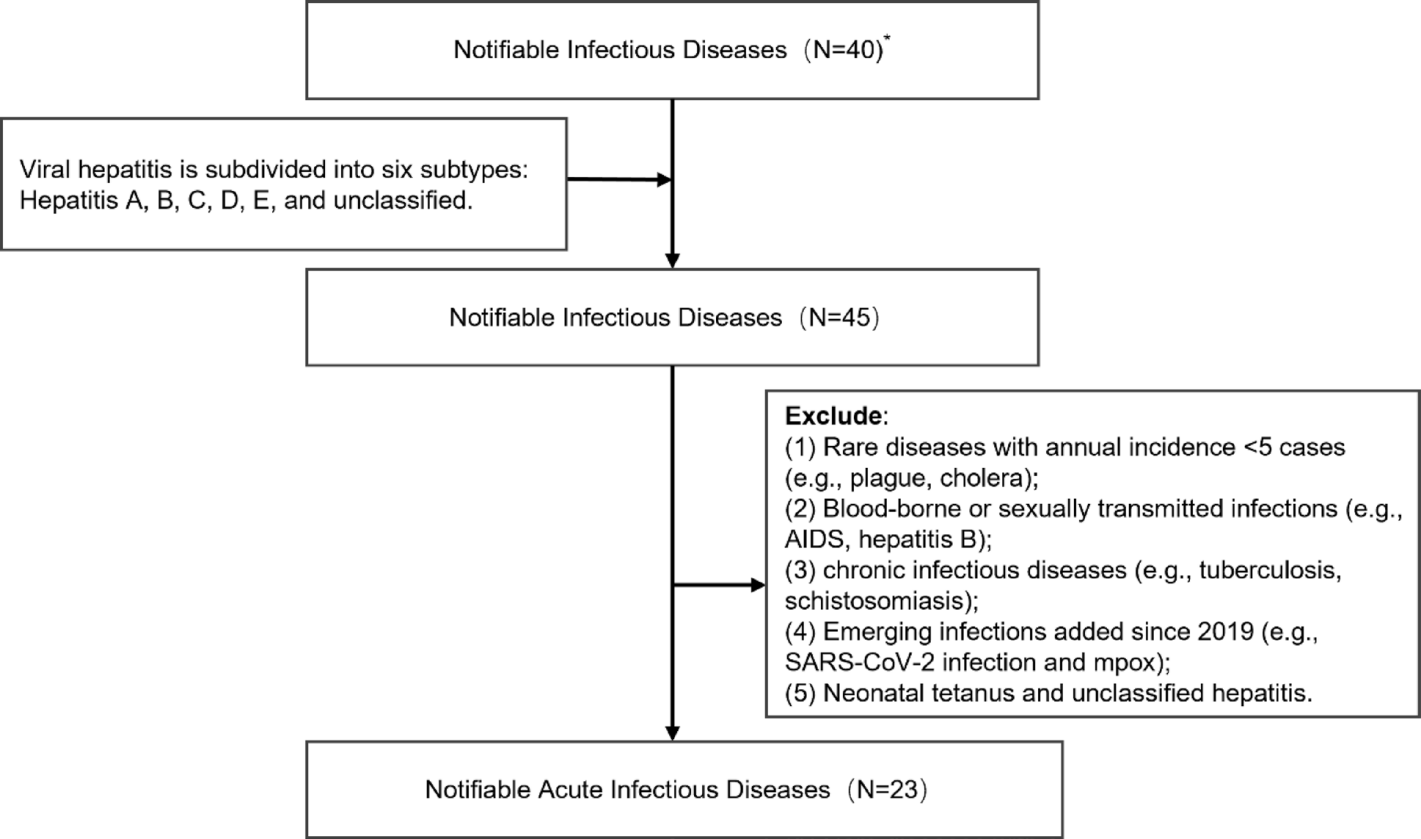
Flowchart of Inclusion–Exclusion for Notifiable Infectious Diseases in Sichuan Province. *: Totally 40 infectious diseases were included in the new China‘s Law on the Prevention and Control of Infectious Diseases [20]

China’s national surveillance system monitors 40 notifiable infectious diseases, categorized into A, B, and C [20]. We refined the viral hepatitis classification by subdividing it into six subtypes (hepatitis A, B, C, D, E, and unclassified), expanding the total monitored diseases from 40 to 45. From this list, we selected 23 major acute infectious diseases (Fig. 1).

The 23 acute infectious diseases selected for this study are characterized by an abrupt onset, a tendency to cause outbreaks, and the need for immediate public health intervention and control measures. Occasional chronic courses may occur in specific diseases, such as bacillary dysentery and typhoid fever, but these are exceptions to their acute nature [21–23]. In this study, infectious diarrhea refers to other infectious diarrhea among the legally reportable infectious diseases, excluding cholera, bacterial and amoebic dysentery, and typhoid and paratyphoid fever.

#### Diseases classification by transmission route

Based on previous studies and official classifications from the Chinese Center for Disease Control and Prevention (China CDC) [8], we categorized the 23 diseases into four transmission-based groups: respiratory diseases (7): measles, meningococcal meningitis, pertussis, scarlet fever, seasonal influenza, mumps, rubella; intestinal diseases (5): hepatitis A, hepatitis E, bacterial and amoebic dysentery, typhoid and paratyphoid, infectious diarrhea; zoonotic and vector-borne diseases (9): hemorrhagic fever, Japanese encephalitis, dengue fever, brucellosis, rabies, anthrax, leptospirosis, malaria, typhus; and diseases with multiple transmission routes (2): acute hemorrhagic conjunctivitis, hand, foot, and mouth disease (HFMD).

#### Study periods

The surveillance periods varied according to national reporting protocols. HFMD data were available from 2008 onward, seasonal influenza from 2013 following the integration of H1N1 into routine surveillance [24], and data on the remaining 21 diseases from 2005 onwards.

### Statistical analysis

We defined incidence and mortality rates (per 100,000 people) as the total number of cases and deaths across all years divided by the total population size. We defined the case-fatality ratio (per 1,000 cases) as the total number of deaths divided by the total number of cases. We used radar diagrams based on monthly case numbers to describe seasonal distribution. Additionally, we divided the ratio of average monthly cases into five levels with a step size of 0.2 to identify acute infectious diseases requiring special attention each month. We also standardized the incidence rate of each infectious disease from 0 to 1 according to percentile rank. We then visualized the annual trend and the standardized data as heat maps.

We used joinpoint regression models to examine incidence, mortality, and case-fatality trends and to identify changes in trends. This method connects several different line segments, providing a concise characterization of changes in trends over time [25]. In this study, we measured temporal trends using age-standardized incidence rates (ASRs) and expressed them as annual percentage changes (APC) and average annual percentage changes (AAPC) between 2005 and 2024. We calculated the AAPC as 100 × (exp(β) − 1) and obtained its 95% confidence interval (CI) from the linear regression model [26]. We used Z-tests to assess whether the AAPC was significantly different from zero. When describing trends, we used the terms “increase” and “decrease” for significant slopes (*p* < 0.05), and “stable” for non-significant average annual percentage changes (*p* ≥ 0.05).

We employed trend surface analysis using the Geostatistical Analyst extension in ArcGIS to describe the spatial structure and patterns of incidence of the 23 acute infectious diseases. A polynomial trend-surface model was fitted to the observed incidence rates based on geographic coordinates (longitude and latitude). This technique approximates the real-world spatial distribution of disease incidence and effectively illustrates the spatial patterns of observed values [27]. The unknown-node incidence rates were spatially interpolated through ordinary least squares (OLS) minimization of squared surface deviations, which produced a three-dimensional projection that highlights regional trends and localized anomalies [28]. In this study, we placed the incidence rates in geospatial coordinates and observed the how the values changed in different directions, particularly in the north-south and east-west orientations. This provided insight into the spatial distribution and directional trends of disease incidence.

We visualized and analyzed disease maps of incidence rates from 2005 to 2024 using trend surface analysis in ArcGIS 10.7.0 (ESRI, Redlands, CA, USA), based on city-level data. Statistical analyses were performed using R version 4.4.1 (The R Foundation for Statistical Computing, Vienna, Austria) and the Joinpoint Regression Program version 5.4.0 (National Cancer Institute, Bethesda, MD, USA). A p-value less than 0.05 was considered statistically significant.

## Results

### Trend of incidence, mortality, and case-fatality ratio of 23 acute infectious diseases

A total of 5,028,418 cases of the 23 major notifiable acute infectious diseases were reported from 2005 to 2024, resulting in an average incidence rate of 305.78 per 100,000 people in Sichuan Province. The total incidence of the 21 diseases under continuous surveillance (excluding HFMD and seasonal influenza) exhibited an increasing trend from 2005 to 2024, with an AAPC of 3.4% (95% CI: 1.75 to 4.51). HFMD showed an increasing trend from 2008 to 2024, with an AAPC of 11.03% (95% CI: 4.62 to 23.37). A rising trend in seasonal influenza was also detected from 2013 to 2024, with an AAPC of 58.59% (95% CI: 41.26 to 128.05).

An analysis of transmission routes revealed an increasing trend in the incidence of respiratory diseases (AAPC: 18.91%, 95% CI: 11.07 to 29.24) and intestinal infectious diseases (AAPC: 13.79%, 95% CI: 2.71 to 25.64). However, the incidence rate of zoonotic and vector-borne diseases demonstrated a downward trend (AAPC: -3.23%, 95% CI: -5.45 to -1.93). Of the 23 acute notifiable infectious diseases, eight showed increasing trends (e.g., pertussis, seasonal influenza, and hepatitis E) and 12 showed decreasing trends (e.g., measles, meningococcal meningitis, and mumps), and three remained stable from 2005 to 2024 (Table 1).

**Table 1:** Trends in the incidence rate of 23 acute infectious diseases in Sichuan from 2005 to 2024

Diseases	Incidence rate (/100 000)		AAPC (%,95%CI)	Trend
	2005	2024		
Additional 21 infectious diseases^†^	152.80	267.77	3.4*(1.75~4.51)	Increase
Respiratory diseases	54.99	919.32	18.91*(11.07~29.24)	Increase
Measles	8.56	0.12	-23.66* (-42.18~-19.58)	Decrease
Meningococcal meningitis	0.15	0.00	-16.49* (-22.11~-12.58)	Decrease
Mumps	38.30	8.00	-5.39* (-8.7~-2.94)	Decrease
Pertussis	0.53	67.12	35.43* (32.43~50.22)	Increase
Rubella	2.46	0.03	-8.16* (-17.52~-3.8)	Decrease
Scarlet fever	2.23	2.92	2.66(-1.17~6.82)	Stable
Seasonal influenza^‡^	2.75	841.12	58.59* (41.26~128.05)	Increase
Intestinal diseases	96.29	187.23	3.79* (2.71~4.64)	Increase
Hepatitis A	10.06	1.49	-11.17* (-12.47~-10.34)	Decrease
Hepatitis E	0.36	3.08	10.06* (7.87~13.95)	Increase
Bacterial and amoebic dysentery	35.54	3.11	-12.81* (-14.43~-11.89)	Decrease
Typhoid and paratyphoid	1.53	0.42	-5.36* (-7.32~-2.99)	Decrease
Infectious diarrhoea^§^	48.79	179.13	7.21* (6.01~8.29)	Increase
Zoonotic and Vector-borne diseases	3.21	1.52	-3.23* (-5.45~-1.93)	Decrease
Anthrax	0.12	0.10	-4.67* (-8.08~-1.97)	Decrease
Brucellosis	0.00	0.56	29.62* (26.85~38.59)	Increase
Dengue fever	0.00	0.11	44.97* (38.53~98.85)	Increase
Hemorrhagic fever	0.34	0.25	-0.97(-4.04~1.68)	Stable
Japanese encephalitis	1.07	0.01	-15.54* (-19.9~-13.53)	Decrease
Leptospirosis	0.95	0.01	-16.7* (-31.09~-10.2)	Decrease
Malaria	0.60	0.25	-4.94* (-9.22~-1.56)	Decrease
Rabies	0.06	0.00	-11.53* (-16.56~-7.32)	Decrease
Typhus	0.07	0.24	7.42* (5.8~10.01)	Increase
Disease with multiple transmission routines	13.39	67.23	28.98* (14.2~63.83)	Increase
HFMD^‡^	12.33	66.42	11.03* (4.62~23.37)	Increase
Acute hemorrhagic conjunctivitis	1.06	0.81	-6.01(-14.52~4.53)	Stable

^†^The additional 21 acute infectious diseases under continuous surveillance exclude HFMD and seasonal influenza. ^§^ Infectious diarrhea refers to other infectious diarrhea among the legally reportable infectious diseases, excluding cholera, bacterial and amoebic dysentery, and typhoid and paratyphoid fever. ^‡^ HFMD data were analyzed starting in 2008, while seasonal influenza monitoring began in 2013.^*^ AAPC or APC differs significantly from 0 at the alpha = 0.05 level

**Fig. 2:**
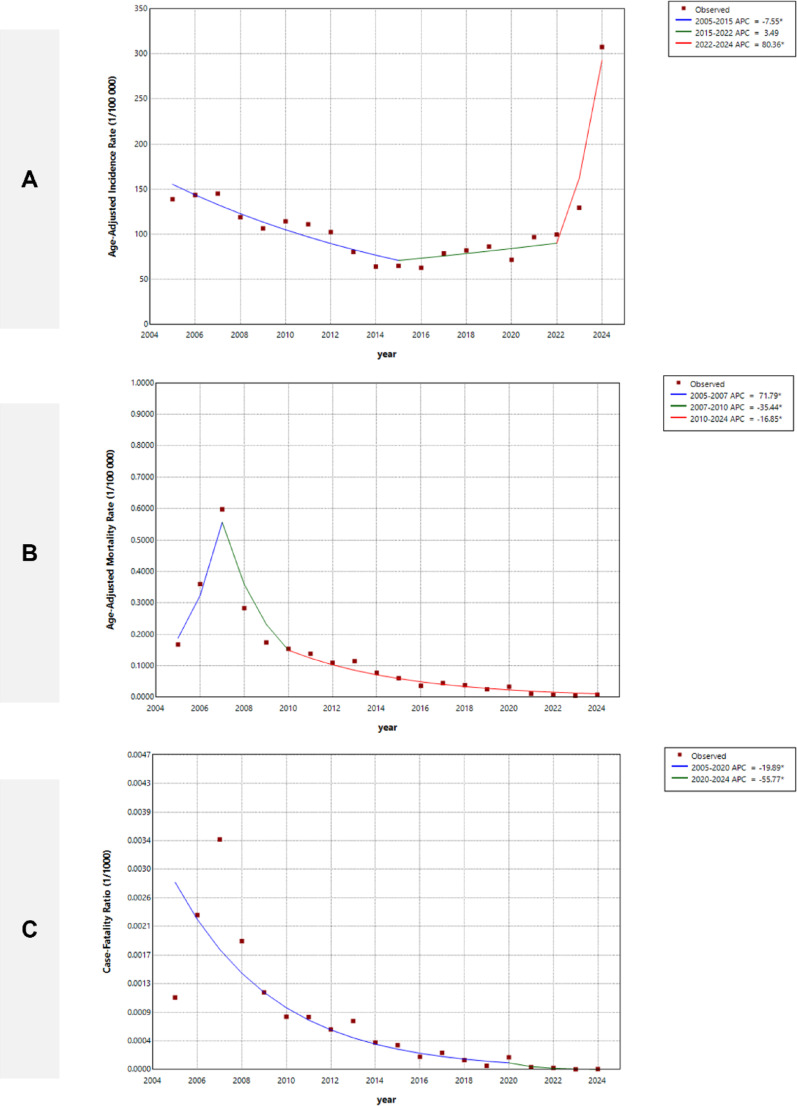
Joinpoint regression showing trends in the overall incidence and mortality rates of the 21 acute infectious diseases from 2005 to 2024 in Sichuan Province, Southwest China. **(A)** Incidence ASR trend for the 21 acute infectious diseases. The final model identified two joinpoints, which divided the trend into three segments: 2005–2015, 2015–2022, and 2022–2024. **(B)** The mortality ASR rate trend for the 21 acute infectious diseases. The final model also identified two joinpoints, resulting in three segments: 2005–2007, 2007–2010, and 2010–2024. **(C)** The case-fatality ratio trend for the 21 acute infectious diseases. The final model selected one joinpoint, dividing the trend into two segments: 2005–2020 and 2020–2024

From 2005 to 2015, the incidence of the 21 acute infectious diseases decreased (APC: -7.55%; 95% CI: -16.58 to -5.02). Then, from 2015 to 2022, there was a stable trend (APC: 3.49%; 95% CI: -3.75 to 16.18). Finally, from 2022 to 2024, there was a sharp increase (APC: 80.36%; 95% CI: 47.33 to 108.31). From 2022 to 2024, pertussis, infectious diarrhea, hepatitis E, brucellosis, and typhus were the top five diseases with the highest APC (Fig. 2, Supporting Information: Table S1).

A total of 2,064 fatalities from 23 major acute infectious diseases were documented over the past two decades. The mortality rate was 0.13 per 100,000 people, and there was a decline in the rate (AAPC: -14.37%; 95% CI: -18.93 to -12.25). From 2005 to 2007, mortality increased (APC: 71.79%; 95% CI: 37.52 to 122.42). Then, it declined from 2007 to 2010 (APC: -35.44%; 95% CI: -42.63 to -23.95) and decreased less pronouncedly from 2010 to 2024 (APC: -16.85%; 95% CI: -22.74 to -2.42).

The case-fatality ratio was 0.41 per 1,000 cases, indicating a consistent downward trend (AAPC: -17.47%; 95% CI: -22.09 to -15.36) from 2005 to 2024 in Sichuan Province. It experienced a consistent decline from 2005 to 2020 (APC: -19.89%; 95% CI: -23.93 to -13.50) and from 2020 to 2024 (APC: -55.77%; 95% CI: -78.48 to -38.63). The latter period saw a more pronounced decline (Fig. 2).

### Ranks and changes of incidence and mortality rate of 23 acute infectious diseases

**Fig. 3:**
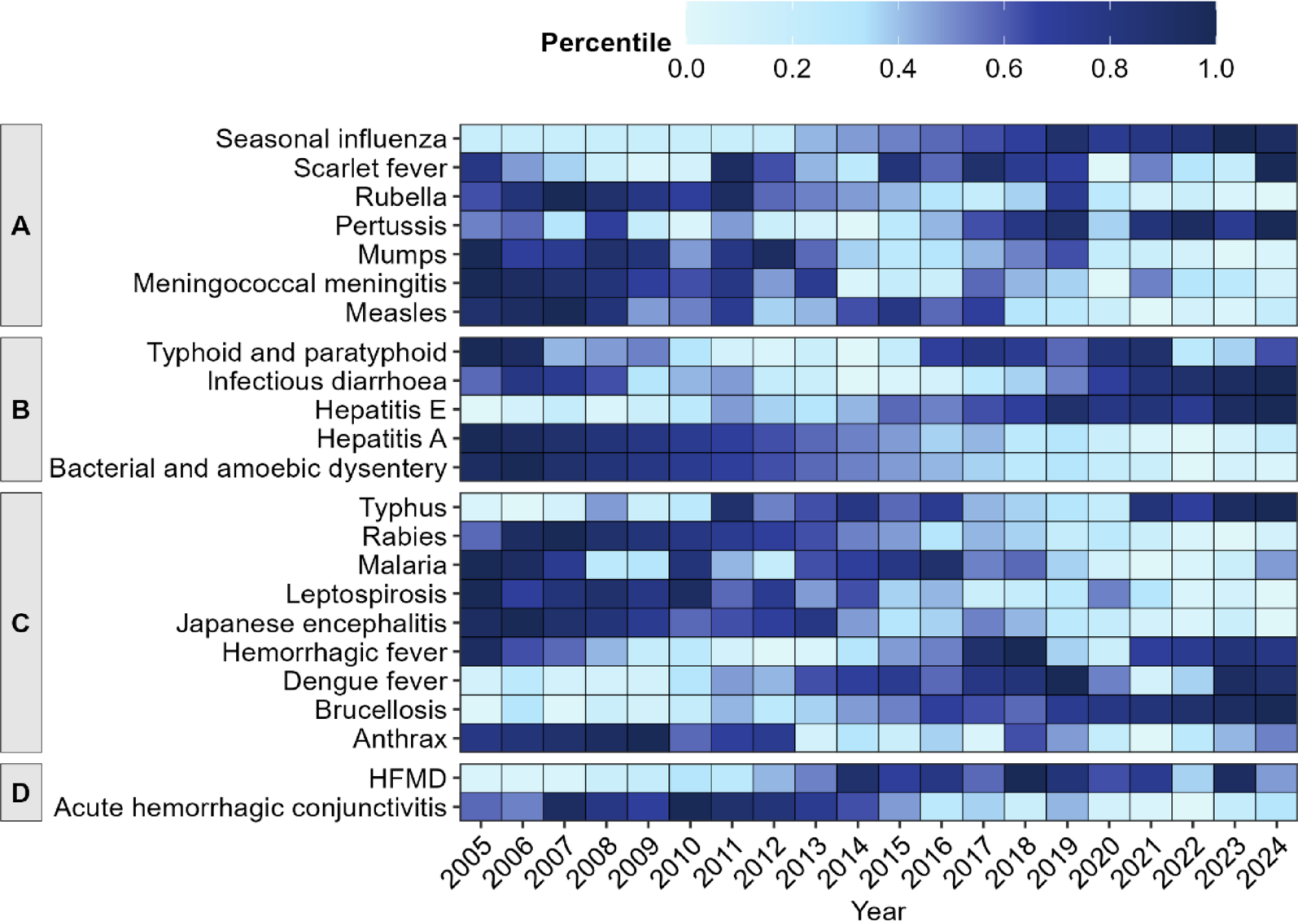
Incidence Rate of 23 Acute Infectious Diseases from 2005 to 2024 in Sichuan, Southwest China. (A) Respiratory infectious diseases. (B) Intestinal infectious diseases. (C) Zoonotic and vector-borne diseases. (D) Diseases with multiple transmission routes. HFMD data were analyzed starting in 2008, while seasonal influenza monitoring began in 2013

From 2005 to 2024, the five acute infectious diseases with the highest incidence rates were seasonal influenza (129.00/100,000 people), HFMD (68.49/100,000 people), infectious diarrhea (55.79/100,000 people), mumps (18.53/100,000 people), and bacterial and amoebic dysentery (13.59/100,000 people). During the first decade, the most prevalent respiratory infectious diseases were measles, meningococcal meningitis, mumps, and rubella. However, seasonal influenza and pertussis have become the most prevalent respiratory infectious diseases in recent years. Among intestinal diseases, hepatitis E and infectious diarrhea have surpassed hepatitis A and bacterial and amoebic dysentery as the leading high-incidence diseases in the last five years. The top five zoonotic and vector-borne diseases by case number from 2005 to 2014 were Japanese encephalitis, malaria, leptospirosis, rabies, and hemorrhagic fever. However, Brucellosis, malaria, hemorrhagic fever, typhus, and dengue fever became the leading five diseases in the past ten years (Fig. 3, Supporting Information: Figure S1, Table S2).

The five acute infectious diseases with the highest mortality rates were rabies, Japanese encephalitis, HFMD, bacterial and amoebic dysentery, and seasonal influenza. During the initial ten-year period, there were 1,784 deaths occurred from the 23 acute infectious diseases. The primary causes were rabies (1,203 deaths, 67.43%), Japanese encephalitis (199 deaths, 11.15%), and HFMD (137 deaths, 7.68%). However, the number of deaths declined significantly in the last decade by 84.31% compared to the previous one. Seasonal influenza replaced Japanese encephalitis in the mortality ranking, with 41 deaths reported in the last decade (14.64% [41/280]).

### Age and sex distribution of incidence rate of 23 acute infectious diseases

**Fig. 4:**
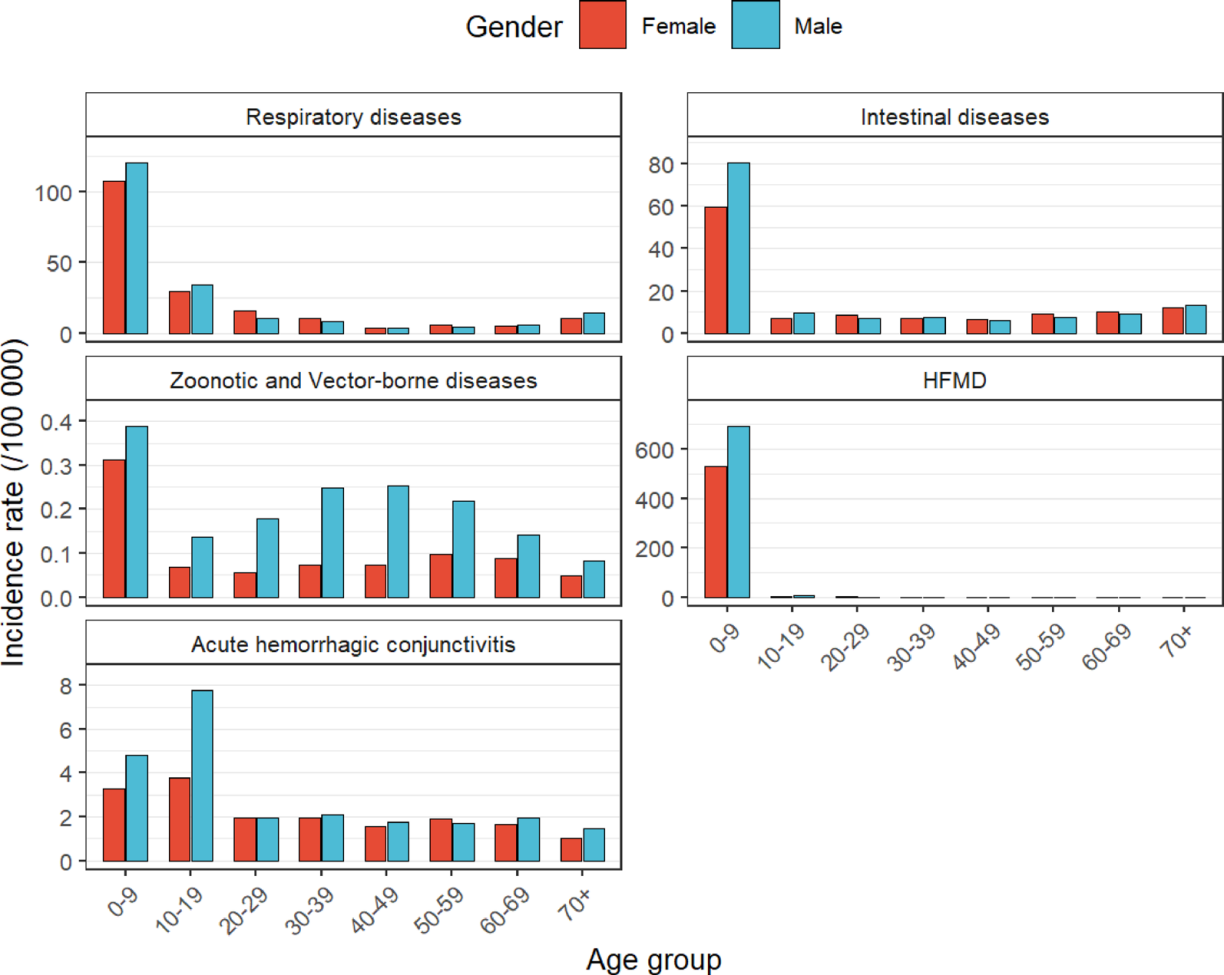
Age-sex Distribution of 23 Acute Infectious Diseases, Stratified by Transmission Route, in Sichuan from 2005 to 2024

From 2005 to 2024, the incidence rates of 23 acute infectious diseases in Sichuan Province exhibited distinct patterns based on gender and age. Overall, the male incidence rate (329.76 per 100,000 people) was higher than the female incidence rate (281.17 per 100,000 people), resulting in a male-to-female incidence ratio of 1.17:1. Zoonotic and vector-borne diseases exhibited significantly higher incidence rates among males in the 20–59 age group, whereas acute hemorrhagic conjunctivitis demonstrated male predominance in age groups under 30 years old. In contrast, respiratory diseases, intestinal diseases, and HFMD showed minimal gender differences (Fig. 4).

An age-stratified analysis revealed considerable heterogeneity. The cohort aged 0–9 years had the highest incidence rate (1,772.04 per 100,000 people), followed by the cohorts aged 10–19 years (276.69 per 100,000 people), 70 + years (150.08 per 100,000 people), 20–29 years (136.38 per 100,000 people), and 30–39 years (106.19 per 100,000 people). Incidence rates remained below 100 per 100,000 people in the 40–69 age group. Compared with other age groups, the 0–9 years cohort had higher incidence rates for HFMD and mumps (Supporting Information: Table S3).

### Geographic distribution and trends of 23 acute infectious diseases

Over the past two decades, the central region of Sichuan Province has had the highest incidence rates of 23 major acute infectious diseases. The five districts with the highest rates were Chengdu, Ya’an, Mianyang, Panzhihua, and A’ba Prefecture. In the first three districts, seasonal influenza was the leading cause, while intestinal infectious diseases, infectious diarrhea, and bacterial and amoebic dysentery were the leading causes in Panzhihua and Aba Prefecture. Consistent with previous results, the geographic distribution of diseases by transmission route showed that respiratory infectious diseases and HFMD were prevalent in the central region centered on Chengdu. Meanwhile, intestinal infectious diseases, as well as zoonotic and vector-borne diseases, were prevalent in the western minority areas of Aba, Liangshan, and Ganzi prefectures (Supporting Information: Figures S2-S4).

**Fig. 5:**
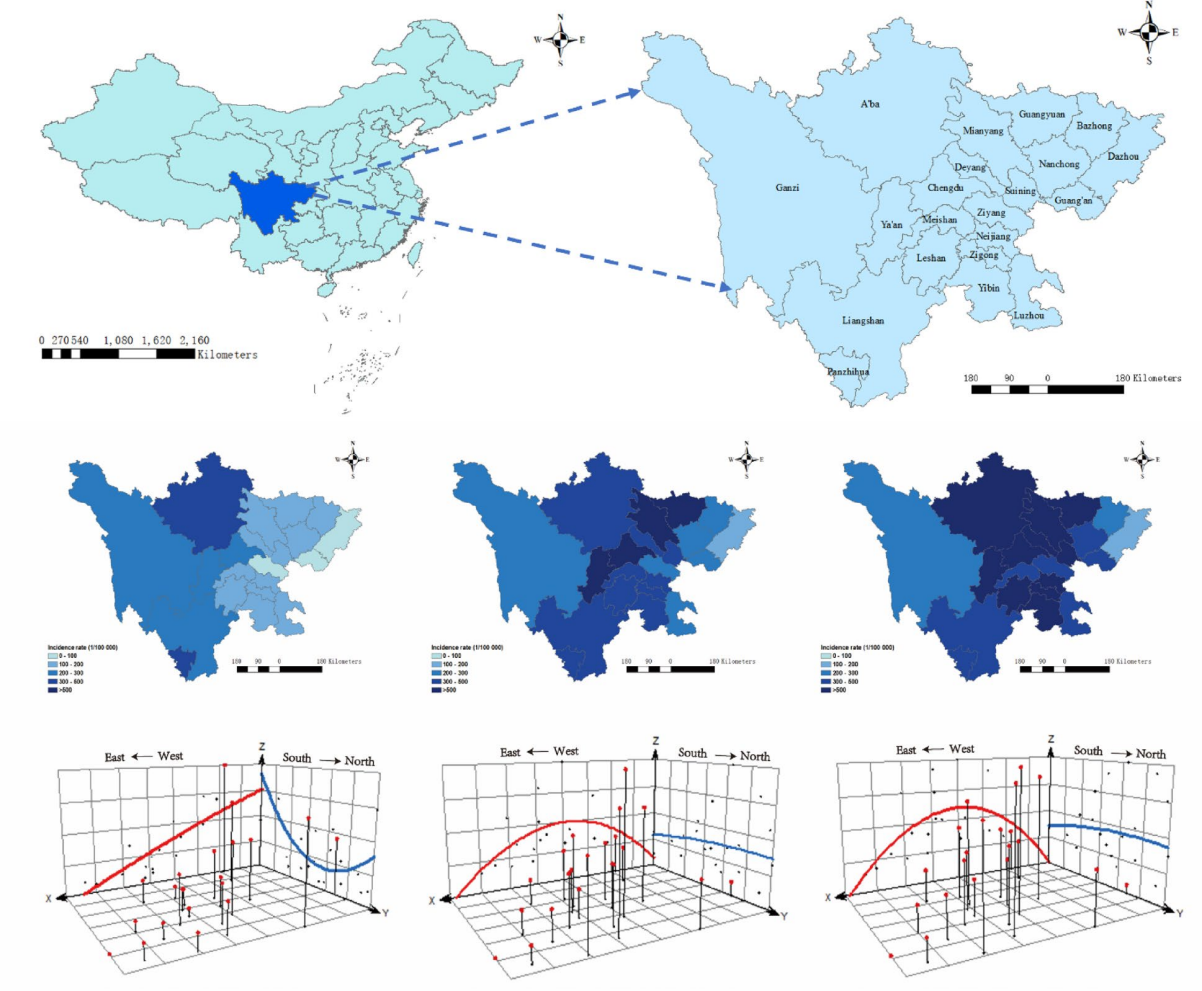
Geographic Distribution and Spatial Trend Analysis of 23 Acute Infectious Diseases in Sichuan Province, Southwest China, Across Three Periods: 2005–2014, 2015–2024, and 2020–2024. Trend surface analysis indicates that the y-axis points north and the x-axis points east, displaying regional incidence rates for 2005–2014, 2015–2024, and 2020–2024, respectively

To capture the dynamic spatial changes of acute infectious diseases, we divided the research period into three stages and conducted a spatial trend surface analysis of Sichuan Province: the first decade (2005–2014), the second decade (2015–2024), and the last five years (2020–2024). Our results revealed that during the first decade (2005–2014), the incidence of 23 acute infectious diseases in the province exhibited a mild U-shaped pattern from north to south, with a higher incidence in the south and a lower incidence in the north. The east-west trend was linear, with a higher incidence in the east and a lower incidence in the west, indicating significant regional disparities. However, the trend surface analysis of the last decade (2015–2024) revealed a different pattern. The north-south incidence pattern became linear, with a slightly higher incidence in the north. Meanwhile, the east-west pattern showed an inverted U-shape: a very low incidence in the eastern and western regions, and a high incidence in the central region. The spatial trend over the last five years (2020–2024) has been similar to that of the last decade but with higher overall incidence levels (Fig. 5).

### Seasonal distribution of 23 acute infectious diseases

**Fig. 6:**
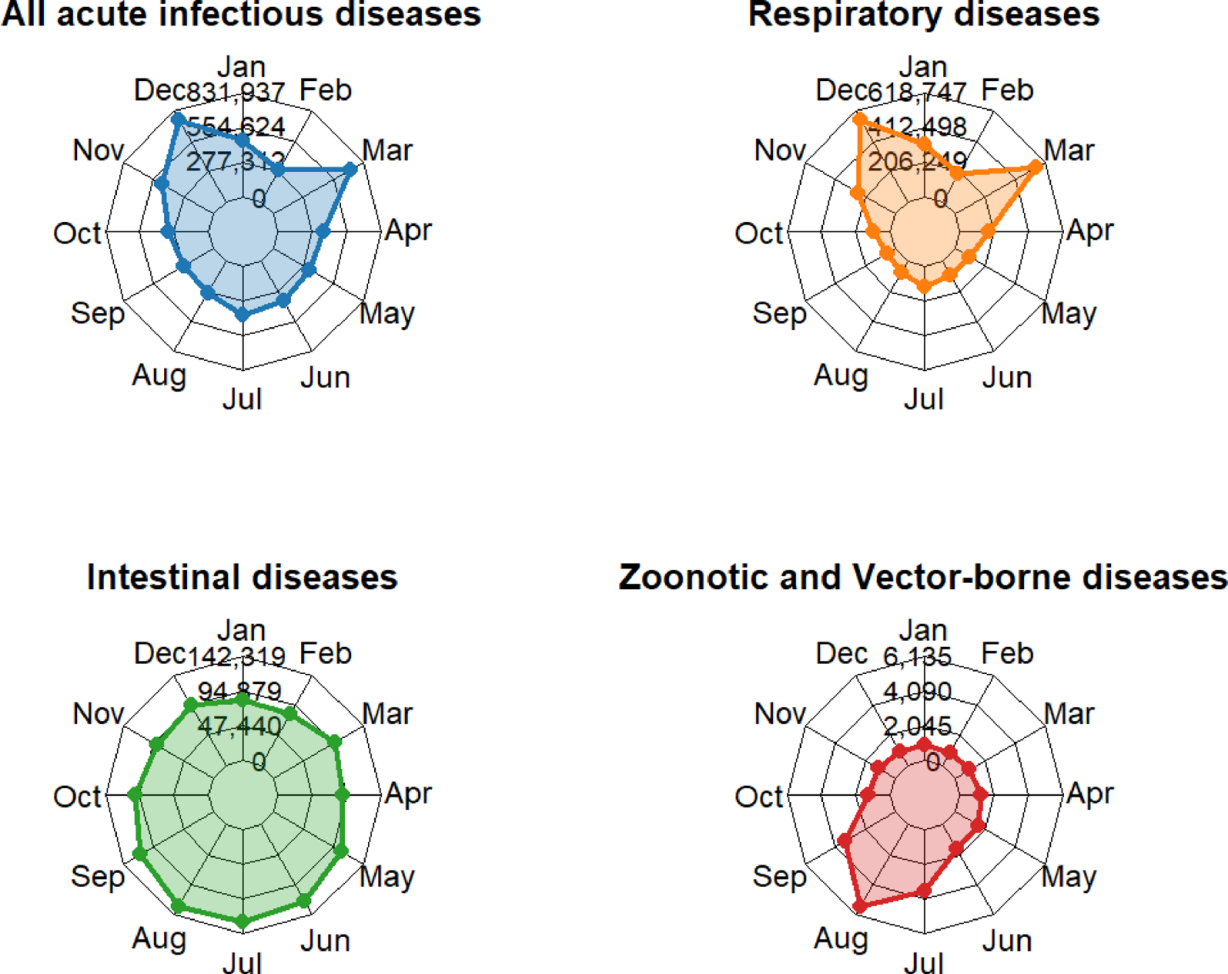
Seasonal Patterns of Different Transmission Routes for 23 Acute Infectious Diseases in Sichuan, from 2005 to 2024. The circumference of the radar diagram represents the 12 months of the year in a clockwise arrangement, and the radius represents the number of incident cases. The diagram shows seasonal trends for respiratory, intestinal, and zoonotic and vector-borne diseases

From 2005 to 2024, peaks in the incidence of 23 acute infectious diseases in Sichuan Province were observed between November and January, as well as in March. The November peak was primarily attributed to seasonal influenza and HFMD, while the other peaks were predominantly linked to seasonal influenza. Additionally, notable seasonal patterns were observed for diseases with various transmission routes. Similar to the overall trend, respiratory infections were most frequently reported between November and January, as well as in March. The incidence of intestinal infectious diseases was slightly higher from May to September. Zoonotic and vector-borne diseases were most prevalent from July to September, during the hottest season of the year (Fig. 6). However, some diseases, such as hepatitis A and E, as well as infectious diarrhea, occurred steadily throughout the year, showing no distinct seasonality (Supporting Information, Figure S5).

## Discussion

To our knowledge, this study is the most comprehensive analysis yet of the epidemiology and trends of acute infectious diseases in Sichuan Province in southwest China. The study has significant public health implications. Following the global pandemic of SARS-CoV-2, the Chinese government substantially expanded its disease surveillance infrastructure [29]. A nationwide integrated system was implemented that links hospitals with prevention and control institutions [30]. As surveillance methods and technologies evolve, examining infectious disease patterns in Sichuan Province using the current stable network-based direct reporting system is crucial. We excluded 2004 from our analysis because it fell within the transitional period between monitoring systems. This ensures a more reliable foundation for future research in this field.

Over the past two decades, the reported incidence of 23 notifiable acute infectious diseases in Sichuan Province has followed a U-shaped trend. A notable increase was observed from 2022 to 2024, primarily driven by rising respiratory and intestinal infectious disease rates. Our findings indicate that overall mortality and case-fatality rates have decreased, consistent with the results reported by N Zhao et al. [8, 31]. The primary contributors to mortality were zoonotic and vector-borne infectious diseases, such as rabies and Japanese encephalitis. As the incidence of these diseases declined, so did the mortality rate. This reduction in deaths from acute infectious diseases is closely linked to the strengthened disease control and prevention measures that China implemented following the 2003 SARS outbreak, as well as the ongoing improvements in the capacity to treat severe infectious disease cases clinically. This progress marks a significant milestone in China’s infectious disease prevention and control efforts [32, 33].

Our study revealed continuous upward trends in pertussis and seasonal influenza. These diseases have replaced traditional acute respiratory infections, such as measles, rubella, and mumps, as the predominant respiratory illnesses. Since the implementation of the measles elimination program, reported incidence rates of measles and rubella in China have remained consistently low [34, 35]. The World Health Organization (WHO) and the Chinese Preventive Medicine Association suggest that the initial rise in pertussis cases is primarily associated with vaccination schedules and outdated vaccine manufacturing processes [36, 37]. However, the sharp increase in pertussis cases observed in recent years may be attributed to several factors. These include the accumulation and evolution of *Bordetella pertussis* pathogenic characteristics, a shift in peak incidence toward older children, heightened surveillance sensitivity among medical staff, improved detection and diagnostic methods, and the broader impact of the COVD-19 pandemic [38–41].

Similarly, viral evolution and low vaccination rates in China have contributed to repeated seasonal influenza epidemics [42, 43]. Changes in seasonal influenza diagnostic criteria in 2019, coupled with the immunity gap created during the COVID-19 pandemic, have facilitated the emergence of new epidemics in China [44–47]. These factors collectively create conditions conducive to sustained increases in seasonal influenza incidence. Studies have found that the global incidence of influenza-dominated respiratory infections is expected to continue rising between 2022 and 2050 [48]. Therefore, further in-depth analysis of influenza risk factors is needed to strengthen management of key areas and high-risk populations and reduce the disease burden.

Contrary to the findings of the other studies [47, 49], infectious diarrhea diseases in Sichuan Province were not effectively controlled during the implementation of non-pharmaceutical interventions. Following the lifting of these measures, there was a steep increase in incidence. This phenomenon may be attributed to disparities in economic development, dietary customs, and food and water safety mechanisms across Sichuan Province’s ethnic regions [50].

Xiang Ren et al. reported that hepatitis A exhibited a downward trend, whereas hepatitis E showed an upward trend in China prior to 2014 [51], which aligns with our findings. Our results indicate a higher incidence of hepatitis E in spring and early summer, with peak attack rates among individuals aged 50–65 years. We hypothesize that the increase in hepatitis E cases is driven by a combination of factors, including higher temperatures and increased precipitation in the spring, as well as the resumption of agricultural activities after the pandemic, which may expose farmers to contaminated water sources [52–54].

Despite the overall decline in zoonotic and vector-borne infectious diseases, brucellosis and typhus have exhibited continuous upward trends in recent years. As early as 2020, the geographic scope of brucellosis cases in Sichuan Province were observed to be expanding geographically [55, 56]. We attribute the sustained increase in brucellosis to two primary factors. First, the development of animal husbandry and changing dietary habits have led to increased consumption of beef and mutton, driving greater interprovincial livestock trade and potentially increasing the number of imported infectious sources. Second, as a non-endemic area for brucellosis, Sichuan Province has limited awareness and capacity for diagnosis and treatment, resulting in delayed case detection that hinders epidemic prevention and control [55, 57]. Following the COVID-19 pandemic, brucellosis incidence has continued to rise by nearly 30% annually. To mitigate brucellosis transmission, we recommend enhancing public health education among the general population and high-risk groups, improving physicians’ diagnostic awareness, strengthening laboratory diagnostic capabilities, and enforcing quarantine and inspection protocols for imported livestock.

Typhus is a frequently overlooked disease that was historically prevalent in regions south of the Yangtze River, including Sichuan Province. The recent increase in cases may be due to greater awareness of the disease and improved diagnostic tools, resulting in more cases being reported. Additionally, climate change, urbanization, and globalization may be expanding the geographic range of rodents carrying infected chiggers [58–60]. Yao Zhang et al. found that typhus in Sichuan Province exhibits distinct seasonality and clustering, with frequent outbreaks occurring in Panzhihua City and Liangshan Prefecture during the summer and fall each year [61]. We recommend implementing targeted control measures during epidemic seasons to reduce rodent and chigger density in high-incidence areas. Furthermore, we recommend strengthening personal protection and health education for travelers entering endemic regions.

Dengue fever is a mosquito-borne infectious disease transmitted by *Aedes aegypti* or *Aedes albopictus*, primarily found in tropical and subtropical regions of Africa, Southeast Asia, the Pacific Ocean, the western Mediterranean, and South America [62]. The first local case of dengue fever occurred in Sichuan Province in 2019, resulting in an outbreak [63]. Our study found that the incidence of dengue fever has decreased since 2019. however, the risk of dengue fever in our province cannot be underestimated. The main reasons are as follows: first, the continuous increase of the floating population and long-term commuting of migrant workers from Southeast Asia; second, some cities along the Yangtze River in our province have climate and geographical conditions that are very suitable for breeding *Aedes albopictus* [64]; and third, medical institutions in our province lack awareness and technology for diagnosing and treating cases [63]. Therefore, we propose increasing training for clinical and public health doctors, improving awareness of diagnosis and treatment, and enhancing surveillance sensitivity to prevent local dengue cases and outbreaks.

Geographic distribution and trend surface analysis revealed that disease occurrence intensity varies across different regions. The incidence of 23 acute infectious diseases is concentrated primarily in the central region, with lower rates observed along the eastern and western boundaries. Respiratory infectious diseases and HFMD are the predominant illnesses in the central region, which is centered around Chengdu. This economically developed area has a high population density, which facilitates the occurrence and spread of respiratory infections [65, 66]. In contrast, intestinal infectious diseases and zoonotic and vector-borne diseases are more prevalent in the western region, particularly in Aba, Ganzi, and Liangshan Prefectures. These economically less developed areas face suboptimal sanitary conditions, limited access to healthcare, and inadequate public health programs and infrastructure, all of which contribute to the transmission of intestinal infectious diseases. Notably, the Panxi region, which includes Panzhihua and Liangshan Prefecture, has a mild climate, averaging around 20 °C year-round. This environment is conducive to vector and rodent breeding, which further facilitates the spread of zoonotic and vector-borne diseases [61]. Therefore, targeted disease prevention and control measures tailored to specific geographical characteristics are essential to reducing the burden of acute infectious diseases in Sichuan Province.

This study identified significant seasonal patterns among different disease categories. Respiratory diseases were prevalent in the winter and spring, intestinal infectious diseases were prevalent in the summer and fall, and zoonotic and vector-borne infectious diseases peaked in the summer. These findings highlight acute infectious diseases requiring particular attention during specific months, providing valuable guidance for public health interventions. However, following the onset of the SARS-CoV-2 pandemic, some infectious diseases that typically occur seasonally have exhibited atypical seasonal variations and elevated incidence rates [41, 44]. Therefore, longer-term observational studies and more precise analyses integrating both seasonal trends and geographical distribution are necessary to establish a reliable scientific basis for disease prevention and control.

## Limitations

This study has several limitations that should be acknowledged. First, since the data were derived from passive surveillance systems, the quality of reporting significantly impacted the accuracy of the data and may have introduced systematic bias. At the same time, mortality data should be interpreted with caution due to known reporting limitations and serve primarily as supplementary indicators for burden estimation rather than definitive fatality measures. Second, the pandemic substantially influenced disease trends. The continuous analysis of data during this period may not accurately reflect true epidemiological patterns throughout the entire study period because certain disease characteristics may have been distorted by pandemic-related disruptions. Third, Sichuan Province has a complex geographical and climatic landscape and considerable diversity among its indigenous ethnic groups. Further in-depth research is needed to investigate disease characteristics across different geographic regions and develop more precise prevention and control strategies.

## Conclusions

The patterns of acute infectious diseases in Sichuan have continuously shifted due to evolving prevention strategies and epidemiological trends. Enhanced surveillance is required for priority diseases, including seasonal influenza, pertussis, infectious diarrhea, hepatitis E, brucellosis, typhus, and dengue fever. Reducing the disease burden effectively requires targeted strategies that integrate seasonal and regional patterns and intensify surveillance during high-risk periods.

## Supplementary Information

Below is the link to the electronic supplementary material.

Supplementary Material 1

## Data Availability

No datasets were generated or analysed during the current study.

## Abbreviations

LMICs: low- and middle-income countries
MERS: Middle East Respiratory Syndrome
SARS: Severe Acute Respiratory Syndrome
COVID-19: Corona Virus Disease 2019
CISDCP: China Information System for Disease Control and Prevention
HFMD: Hand, Foot, and Mouth Disease
CDC: Centers for Disease Control and Prevention
ASRs: Age-Standardized Incidence Rates
APC: Annual Percentage Changes
AAPC: Average annual percentage changes
CI: confidence interval
OLS: ordinary least squares
WHO: World Health Organization
