# Supplementary material for: Epidemiological shifts in 23 acute infectious diseases in Southwest China (2005–2024): trends and implications for surveillance
Source: BMC Public Health. 2025 Sep 24;25:3098. doi: 10.1186/s12889-025-24390-9 (PMC12462255; doi:10.1186/s12889-025-24390-9)

**Table S1.** Joinpont regression analysis results of all the 23 acute infectious diseases in Sichuan Province, Southwest China, from 2005 to 2024

^†^ The additional 21 acute infectious diseases under continuous surveillance exclude HFMD and seasonal influenza. ^§^ Infectious diarrheal diseases other than cholera, bacterial and amoebic dysentery, and typhoid and paratyphoid fever. ^‡^ HFMD data were analyzed starting in 2008, while seasonal influenza monitoring began in 2013.

**Table S2.** The top ten acute infectious diseases with the highest incidence rate across three periods: 2005–2014, 2015–2024, and 2020–2024 in Sichuan Province, Southwest China.

**Table S3.** The top five acute infectious diseases with the highest incidence rate in different age-groups in Sichuan Province, Southwest China, from 2005 to 2024.

**
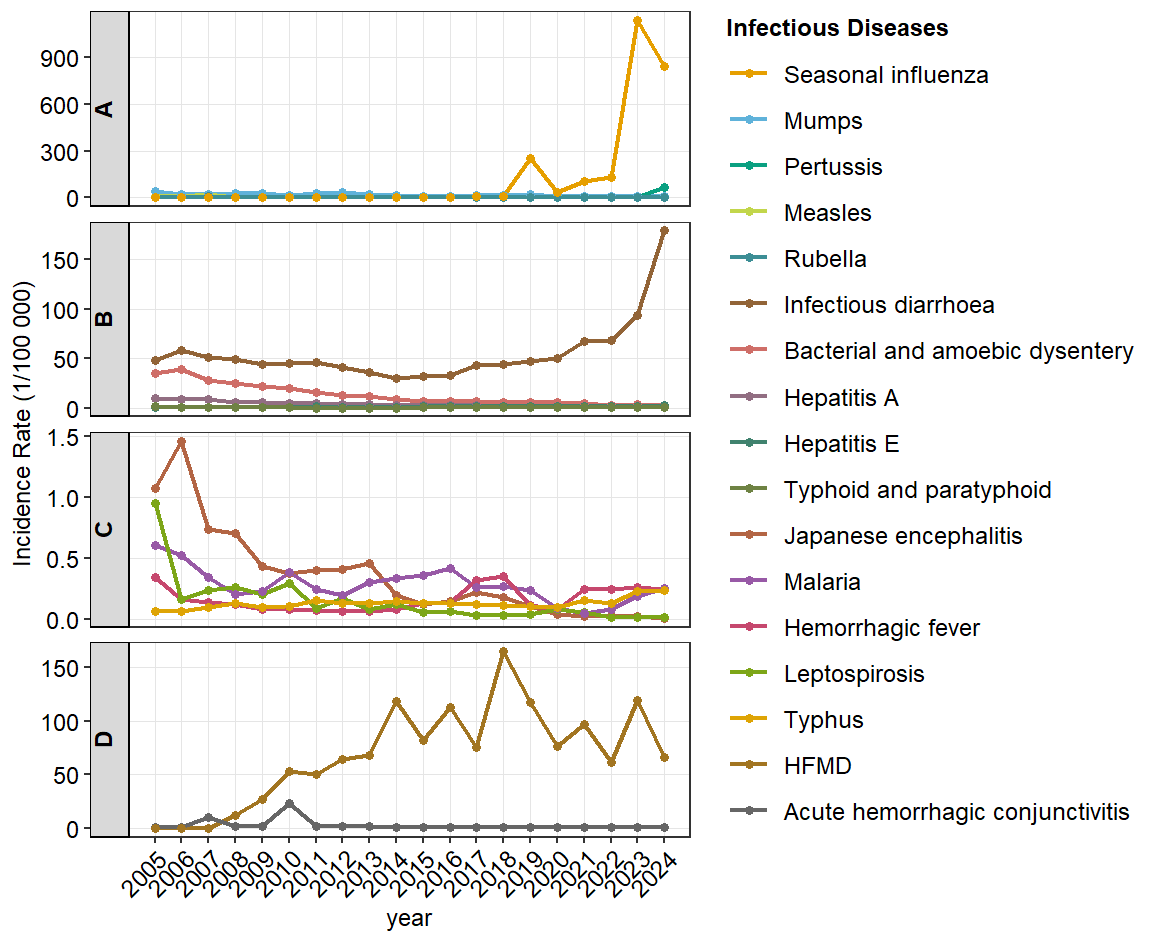
Figure S1.** Incidence rates of the top five diseases by transmission route in Sichuan Province, Southwest China, from 2005 to 2024.

(A) Respiratory infectious diseases. (B) Intestinal infectious diseases. (C) Zoonotic and vector-borne diseases. (D) Diseases with other transmission routes.

**
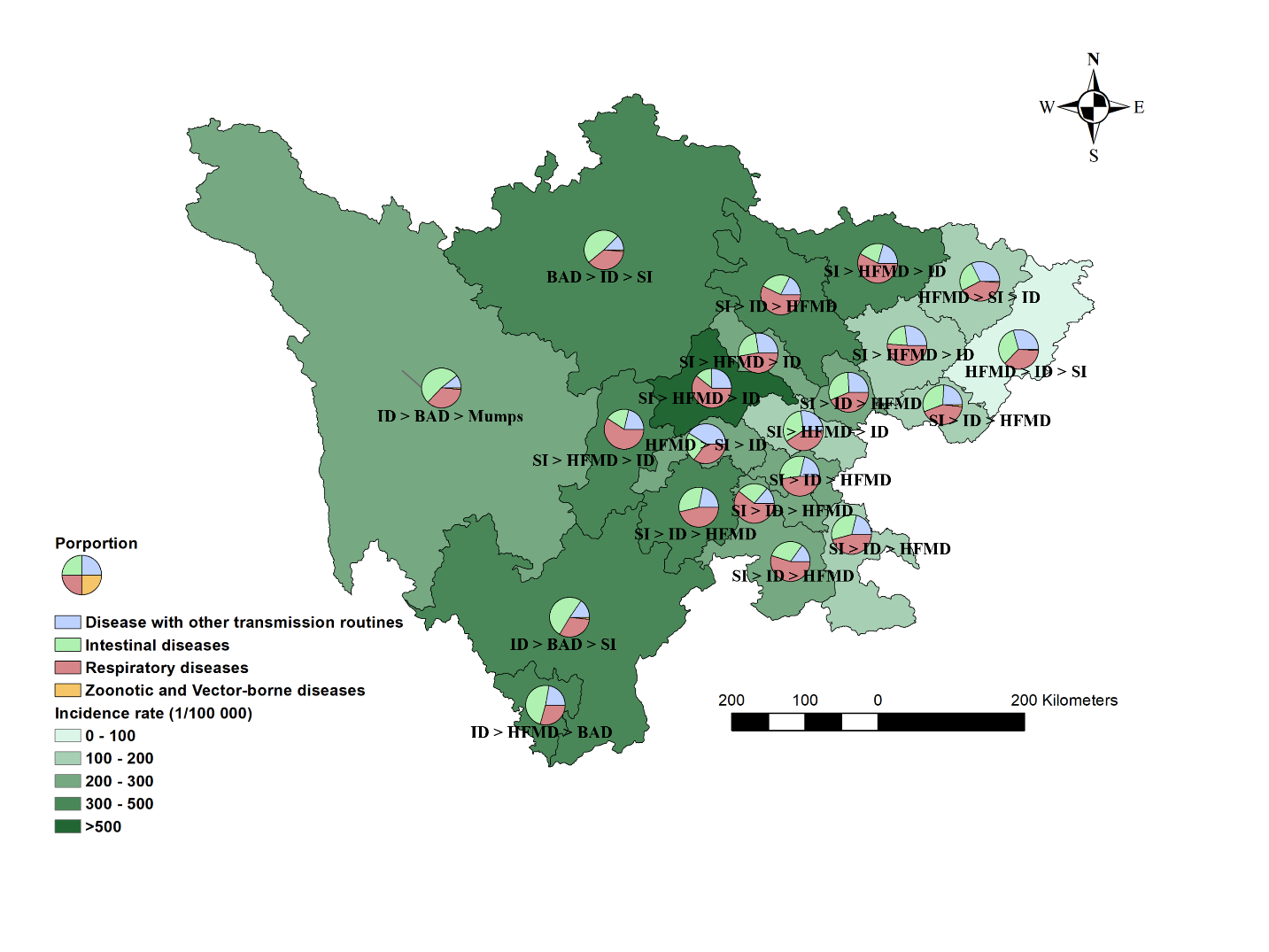
Figure S2.** Geographic distribution of incidence rate, composition by different transmission routes, and the top three diseases of 23 acute infectious diseases in Sichuan Province, Southwest China, from 2005 to 2024.

HFMD, hand, foot, and mouth disease; SI, Seasonal influenza; ID, Infectious diarrhea; BAD, bacterial and amoebic dysentery.

**Figure S3.** Geographic distribution of incidence rate of each 23 acute infectious diseases in Sichuan Province, Southwest China, from 2005 to 2024.


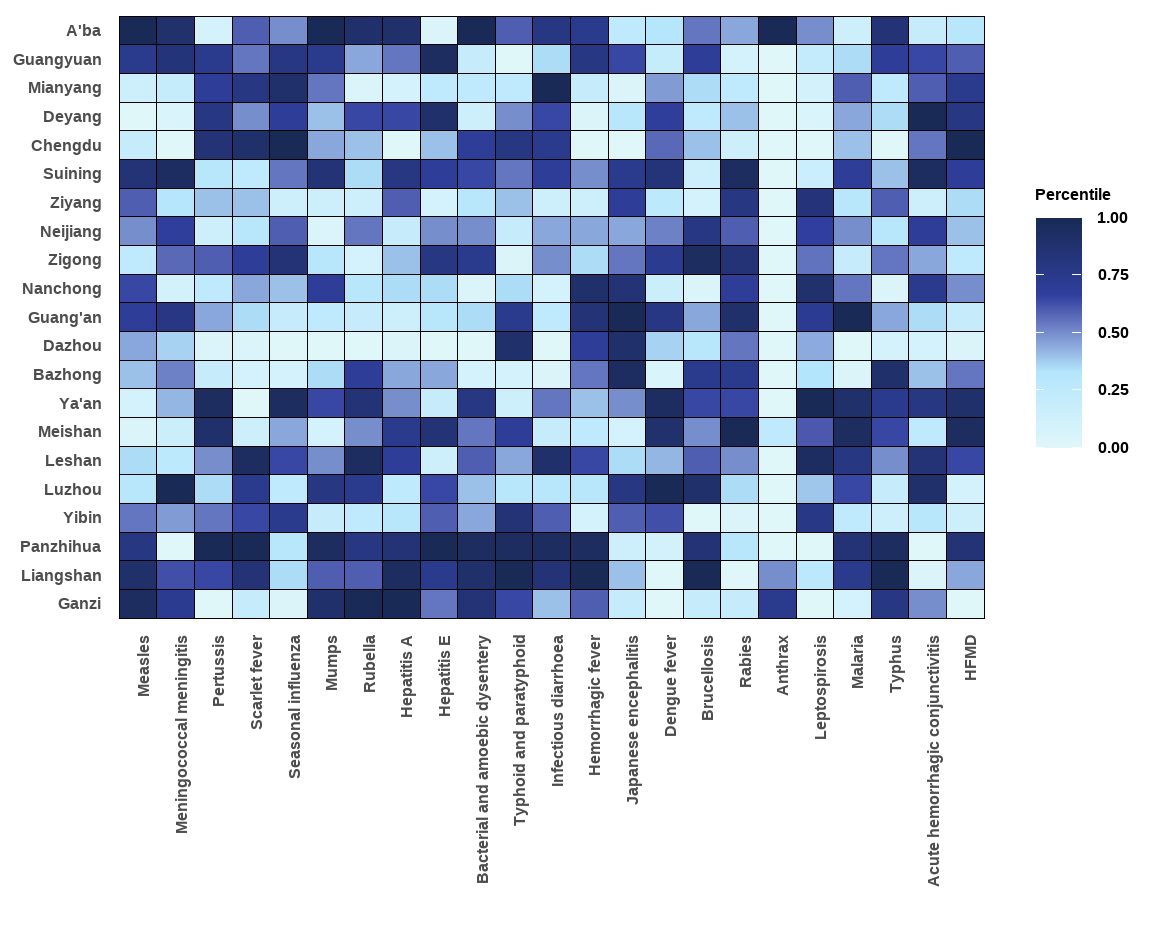


**Figure S4.** Geographic distribution of average incidence rate by different transmission routes of 23 acute infectious diseases in Sichuan Province, Southwest China, from 2005 to 2024.

**
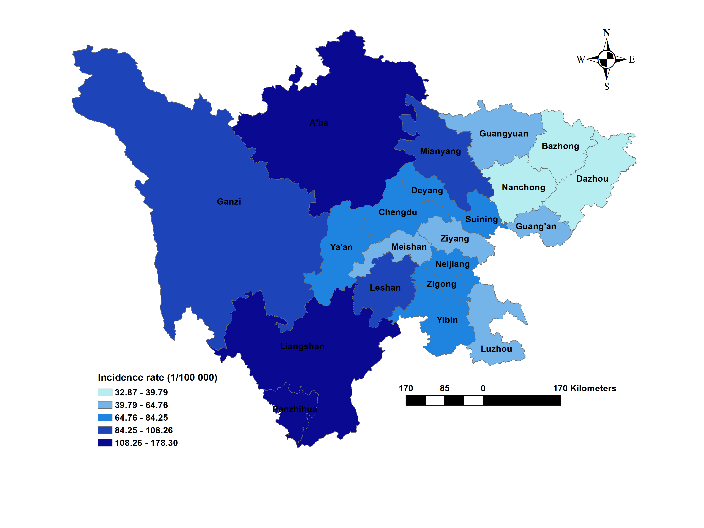

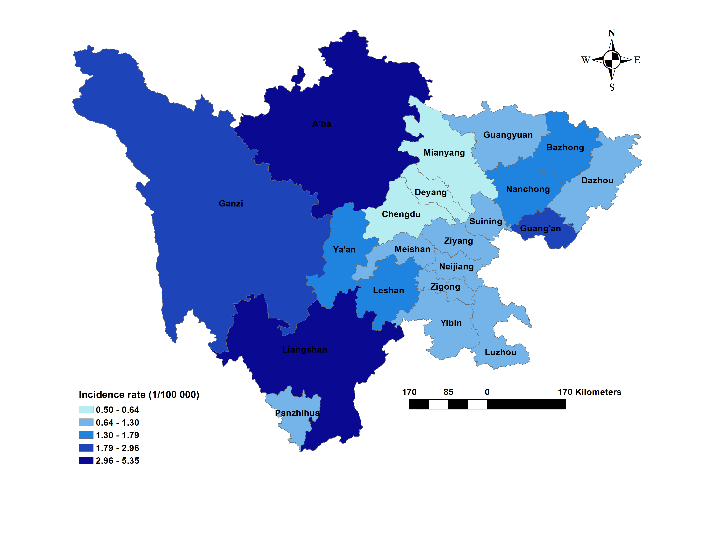

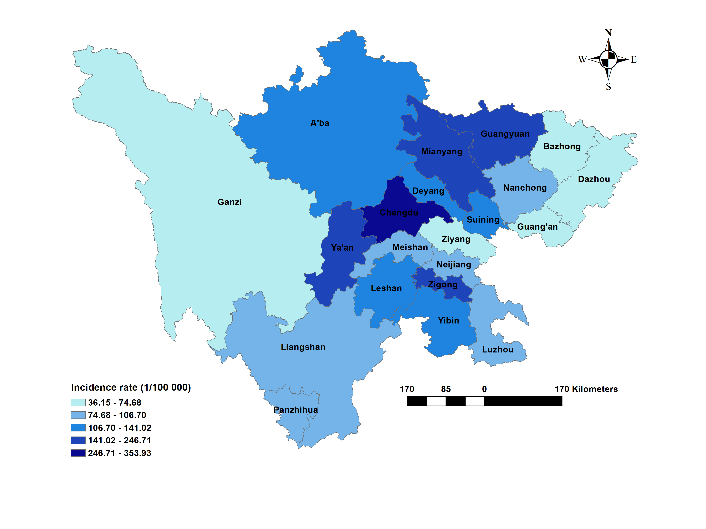
**

**Respiratory diseases Intestinal diseases Zoonotic and vector-borne diseases**

**
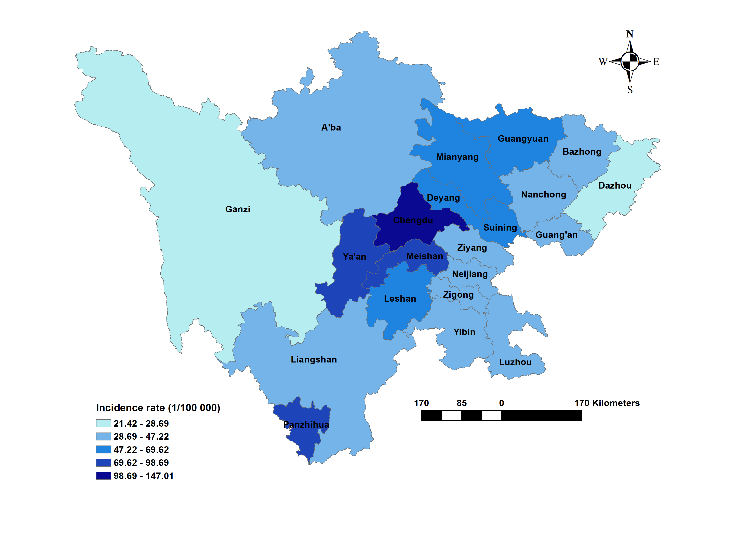

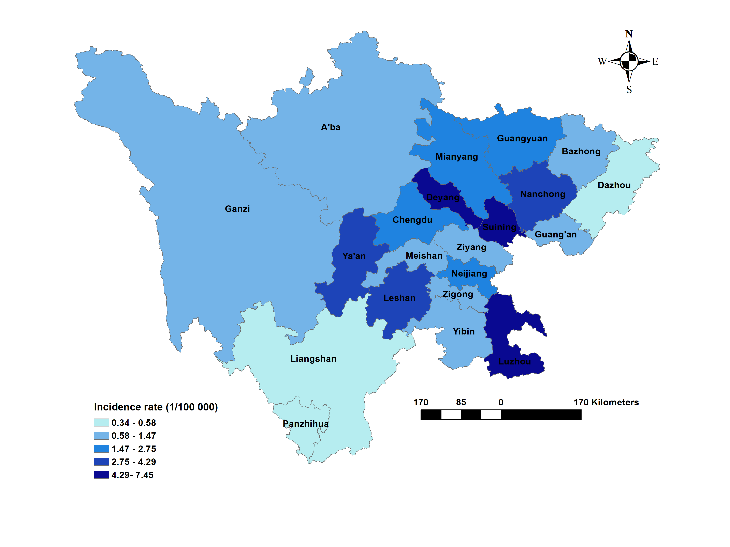
**

**HFMD Acute hemorrhagic conjunctivitis**

**Figure S5.** Heat map of 23 acute infectious diseases monthly incidence rate in Sichuan Province, Southwest China, from 2005 to 2024.


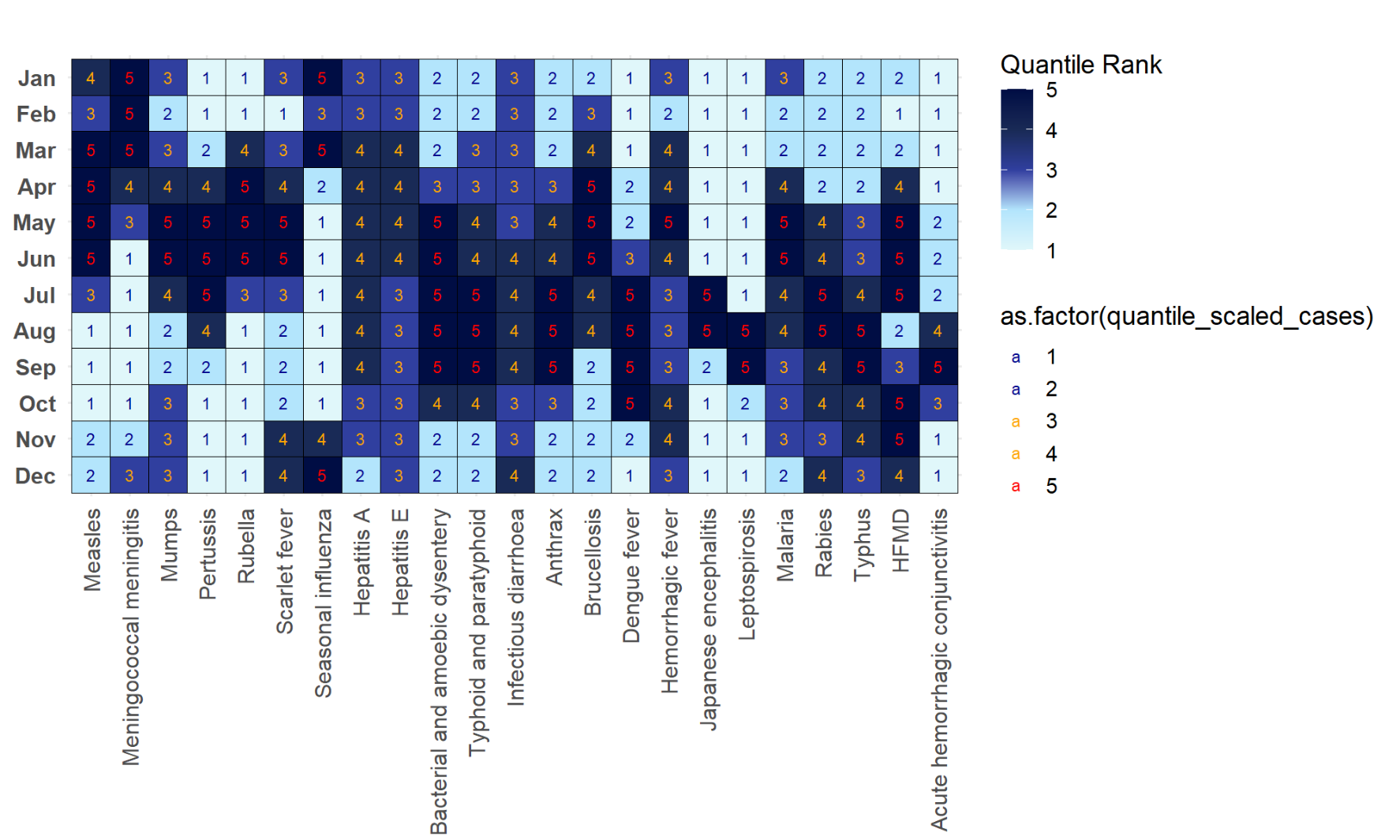

Supplement: Supplementary file 1 — Supplementary Material 1 [file 12889_2025_24390_MOESM1_ESM.docx]
